# Trilaciclib prior to chemotherapy reduces the usage of supportive care interventions for chemotherapy‐induced myelosuppression in patients with small cell lung cancer: Pooled analysis of three randomized phase 2 trials

**DOI:** 10.1002/cam4.4089

**Published:** 2021-08-18

**Authors:** Renata Ferrarotto, Ian Anderson, Balazs Medgyasszay, Maria Rosario García‐Campelo, William Edenfield, Trevor M. Feinstein, Jennifer M. Johnson, Sujith Kalmadi, Philip E. Lammers, Alfredo Sanchez‐Hernandez, Yili Pritchett, Shannon R. Morris, Rajesh K. Malik, Tibor Csőszi

**Affiliations:** ^1^ University of Texas MD Anderson Cancer Center Houston TX USA; ^2^ St Joseph Heritage Healthcare Santa Rosa CA USA; ^3^ Veszprém County Lung Medicine Institute Veszprém Hungary; ^4^ University Hospital A Coruña A Coruña Spain; ^5^ Prisma Health Cancer Institute Greenville SC USA; ^6^ Piedmont Cancer Institute Atlanta GA USA; ^7^ Thomas Jefferson University Hospital Philadelphia PA USA; ^8^ Ironwood Cancer & Research Center Chandler AZ USA; ^9^ Baptist Cancer Center Memphis TN USA; ^10^ Hospital Provincial de Castellon Castelló Spain; ^11^ G1 Therapeutics, Inc. Research Triangle Park NC USA; ^12^ Hetenyi Geza Korhaz Szolnok Hungary; ^13^ Present address: Istari Oncology Morrisville NC USA

**Keywords:** anemia, erythropoiesis‐stimulating agent, granulocyte colony‐stimulating factor, neutropenia, red blood cell transfusion, trilaciclib

## Abstract

**Background:**

Supportive care interventions used to manage chemotherapy‐induced myelosuppression (CIM), including granulocyte colony‐stimulating factors (G‐CSFs), erythropoiesis‐stimulating agents (ESAs), and red blood cell (RBC) transfusions, are burdensome to patients and associated with greater costs to health care systems. We evaluated the utilization of supportive care interventions and their relationship with the myeloprotective agent, trilaciclib.

**Methods:**

Data were pooled from three independent randomized phase 2 clinical trials of trilaciclib or placebo administered prior to chemotherapy in patients with extensive‐stage small cell lung cancer (ES‐SCLC). The impact of supportive care on the duration of severe neutropenia (DSN), occurrence of severe neutropenia (SN), and occurrence of RBC transfusions on/after week 5 was analyzed across cycles 1–4. Concordance and association between grade 3/4 anemia, RBC transfusions on/after week 5, and ESA administration was also evaluated.

**Results:**

The use of G‐CSFs, ESAs, or RBC transfusions on/after week 5 was significantly lower among patients receiving trilaciclib versus placebo (28.5% vs. 56.3%, *p* < 0.0001; 3.3% vs. 11.8%, *p* = 0.0254; and 14.6% vs. 26.1%, *p* = 0.0252, respectively). Compared with placebo, trilaciclib significantly reduced DSN and SN, irrespective of G‐CSF administration. RBC transfusions and ESAs were most often administered in patients with grade 3/4 anemia; however, patients typically received RBC transfusions over ESA administration.

**Conclusions:**

By improving CIM and reducing the need for associated supportive care, trilaciclib has the potential to reduce the burden of myelosuppression on patients receiving myelosuppressive chemotherapy for the treatment of ES‐SCLC.

**Trial registration:**

ClinicalTrials.gov (NCT02499770; NCT03041311; NCT02514447).

## Graphical abstract

Compared with placebo, administering trilaciclib prior to chemotherapy reduces chemotherapy‐induced neutropenia and anemia, with a reduction in the use of hematopoietic growth factors and red blood cell transfusions. By improving key myelosuppressive endpoints and reducing the need for associated supportive care, trilaciclib has the potential to reduce both the societal and economic burden of chemotherapy‐induced myelosuppression on patients with extensive‐stage small cell lung cancer.

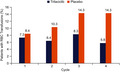

**LAY PERSON SUMMARY**

When people who are treated with chemotherapy develop a condition called myelosuppression, they often receive supportive care treatments that help the body produce new blood cells. However, some supportive care treatments have negative side effects, and many are quite expensive. Researchers found that people who were given a new drug (trilaciclib) before they were treated with chemotherapy did not need as much supportive care as people who were given placebo (an inactive drug). The researchers concluded that trilaciclib may help to reduce the burden of myelosuppression on patients, caregivers, and health care systems.

## 1 INTRODUCTION

Standard chemotherapy‐based regimens for extensive‐stage small cell lung cancer (ES‐SCLC), including carboplatin plus etoposide (E/P), E/P plus atezolizumab (E/P/A), E/P plus durvalumab, and second‐line lurbinectedin and topotecan, often lead to clinically significant myelosuppression.^1^, ^2^, ^3^, ^4^, ^5^ Chemotherapy‐induced myelosuppression (CIM), which commonly manifests as neutropenia, anemia, and thrombocytopenia, is associated with an increased risk of infection, fatigue, and bleeding, all of which can have a profound negative effect on patients’ quality of life.^6^

Chemotherapy‐induced myelosuppression is typically managed with chemotherapy dose delays and/or reductions, along with supportive care involving growth factor administration (granulocyte colony‐stimulating factors [G‐CSFs] or erythropoiesis‐stimulating agents [ESAs]), as well as red blood cell (RBC) and platelet transfusions.^6^, ^7^ Reductions in dose intensity due to dose modifications, dose delays, or chemotherapy discontinuation may compromise treatment efficacy,^8^ and treatment delays can also present a substantial burden to patients, with the need to reschedule visits leading to inefficiencies and greater costs to health care systems.^9^ Moreover, supportive care interventions are lineage specific, are often used reactively, and are each associated with risks and limitations.

For example, although G‐CSFs reduce infectious complications related to febrile neutropenia (FN), their use is commonly associated with bone pain.^10^ RBC transfusions carry risks of occult infection, transfusion reactions, and alloimmunization and present a burden to patients owing to the need for multiple trips to medical facilities and the requirement of blood testing.^11^ ESAs have been associated with serious potential side effects, including an increased risk of cardiovascular events and thromboembolism; accordingly, their use may be restricted to patients with hemoglobin <10 g/dl who are receiving chemotherapy with palliative intent.^12^ Therefore, there is an overall need for additional interventions to reduce the incidence of CIM.

Trilaciclib is an intravenous cyclin‐dependent kinase (CDK)4/6 inhibitor that, when administered prior to chemotherapy, transiently arrests CDK4/6‐dependent hematopoietic stem and progenitor cells (HSPCs) in the G1 phase of the cell cycle during chemotherapy exposure, thus protecting them from chemotherapy‐induced damage (myeloprotection).^13^, ^14^ Unlike HSPCs, SCLC cells replicate independently of CDK4/6 through obligate loss of the retinoblastoma protein.^15^ Consequently, SCLC cells are not arrested by trilaciclib and remain susceptible to the cytotoxic effects of chemotherapy.

The myeloprotective effects of trilaciclib have been investigated in three independent, randomized, placebo‐controlled, double‐blind phase 2 clinical trials in patients with ES‐SCLC, with results demonstrating that administering trilaciclib prior to chemotherapy was effective at reducing the incidence of multilineage CIM. Compared with placebo, administering trilaciclib prior to chemotherapy also resulted in the need for fewer dose modifications, fewer supportive care interventions, and improved quality of life.^16^, ^17^, ^18^, ^19^

In this analysis, data from all three trials were pooled to evaluate the utilization of G‐CSFs, ESAs, and RBC transfusions. The relationship between supportive care interventions and the myeloprotective benefits of trilaciclib was also explored.

## 2 PATIENTS AND METHODS

### 2.1 Study designs

This retrospective analysis used pooled data from the phase 2 portions of three randomized, double‐blind, placebo‐controlled clinical trials in which trilaciclib or placebo was administered prior to chemotherapy (Table S1): G1T28‐05 (NCT03041311; first‐line E/P/A), G1T28‐02 (NCT02499770; first‐line E/P), and G1T28‐03 (NCT02514447; second‐/third‐line topotecan).^16^, ^17^, ^18^ Eligible patients were aged at least 18 years, with confirmed ES‐SCLC, measurable disease per response evaluation criteria in solid tumors (version 1.1), Eastern Cooperative Oncology Group performance status (ECOG PS) 0‒2, and adequate organ function.

In each trial, administration of ESAs and primary prophylaxis with G‐CSFs was prohibited in cycle 1, although therapeutic G‐CSF was allowed. In cycles 2 and higher, supportive care measures, including ESAs and G‐CSFs (prophylactic or therapeutic), were permitted per standard of care guidelines. RBC and platelet transfusions were allowed per investigator discretion throughout the treatment period.

All trials were designed and conducted in accordance with the Declaration of Helsinki and International Council for Harmonization Good Clinical Practice guidelines. The protocols and study‐related materials were approved by the institutional review board or independent ethics committee of each participating site. All patients provided written informed consent.

### 2.2 Endpoints and assessments

Neutrophil and RBC‐related endpoints were assessed, including: duration of severe neutropenia (DSN) in cycle 1 (whereby severe neutropenia [SN] was defined as an absolute neutrophil count [ANC] <0.5 × 10⁹ cells/L), occurrences of SN, G‐CSF administration, grade 3/4 anemia, and ESA administration, and occurrence and number of RBC transfusions on/after week 5. DSN in cycle 1 was defined as the number of days from the date of first ANC value <0.5 × 10^9^/L to the date of first ANC value ≥0.5 × 10^9^/L, with no subsequent ANC values <0.5 × 10^9^/L in that cycle. For patients without SN in cycle 1, DSN was set to 0.

Granulocyte colony‐stimulating factors use was classified as prophylactic (pegfilgrastim or filgrastim), therapeutic (filgrastim), or other (Table 1).

**TABLE 1:** Classification of G‐CSF administration

Category	Definition
Pegfilgrastim	Prophylactic use of long‐acting G‐CSFs, based on a triggering neutropenia event in any previous cycle
Filgrastim	Prophylactic use of short‐acting G‐CSFs (initiated within 3 days of the last chemotherapy dose), based on a triggering neutropenia event in any previous cycle
Therapeutic filgrastim	Therapeutic use of short‐acting G‐CSFs (initiated >3 days after the last chemotherapy dose within the cycle) in a cycle where the patient had a triggering neutropenia event
Other G‐CSF administration	G‐CSFs administered in the absence of any triggering neutropenia event^a^
None	No administration of G‐CSFs

Abbreviations: G‐CSF, granulocyte colony‐stimulating factor; SN, severe neutropenia.

^a^ Occurrence of SN or a neutropenia‐related serious adverse event.

Red blood cell transfusions before week 5 were excluded to ensure that analyses of potential benefit were not confounded by the residual effect of previous treatment. RBC transfusions were considered clinically appropriate for hemoglobin <8.0 g/dl; hemoglobin ≥8.0 and <9.0 g/dl, with a history of atherosclerosis; and/or symptomatic anemia or hospitalization for a life‐threatening event, regardless of hemoglobin value.

### 2.3 Statistical analysis

Data from the three trials were pooled and analyses conducted using intention‐to‐treat (ITT) principles. Treatment effect on DSN in cycle 1 was evaluated using a nonparametric analysis of covariance model, with treatment group difference in mean DSN and its 95% confidence interval (CI) generated using a Satterthwaite *t*‐test. Treatment effect for occurrence of SN and other binary endpoints was assessed using a modified Poisson model with the adjusted relative risk (aRR, trilaciclib vs. placebo), its 95% CI, and two‐sided *p*‐value reported. For endpoints that captured the number of events, treatment group differences were evaluated using a negative binomial regression model, and the aRR, its 95% CI, and two‐sided *p*‐value reported. To account for potential variability among patients and studies when assessing treatment effect, ECOG PS (0/1 or 2), presence of brain metastases (yes or no), and trial (G1T28‐05, G1T28‐02, or G1T28‐03) were used as common factors in all statistical models. Corresponding baseline values were included as covariates where appropriate.

To assess the impact of the potential confounding factor of G‐CSF administration, treatment effect on DSN in cycle 1 and occurrence of SN in cycle 1 were analyzed with an additional term of G‐CSF administration in the model. Occurrence of SN was assessed in cycle 1 rather than across the treatment period because G‐CSF administration was a cycle‐based decision. DSN and occurrence of SN for patients with and without G‐CSF use were also analyzed by cycle for the first four cycles of treatment (longest duration of time shared by most patients enrolled in all three studies). ITT patients were included in the cycle 1 data analysis, while analyses of later cycles only included patients who started the specific cycle. Summary statistics for DSN and occurrence of SN at each cycle for patients with or without G‐CSF administration were provided. The treatment effect on DSN in cycle 1 in each subgroup was tested using the nonparametric model, and the group difference in occurrence of SN in cycles 1 and 2 (most SN occurred in cycle 1 and decreased in subsequent cycles, and G‐CSF administration was associated with treatment cycle) was tested using the modified Poisson model. Consistency of treatment effects on occurrence of SN between patients with or without G‐CSF use at cycles 1 or 2 was tested using a separate modified Poisson model with additional terms of G‐CSF administration and treatment by G‐CSF interaction, whereby statistically significant interaction was defined as *p*_interaction_ <0.20.

The number and percent of patients in each category of G‐CSF use (prophylactic, therapeutic, other) were summarized by treatment group at each cycle.

Subgroup analyses to evaluate the impact of ESA administration on each RBC‐related endpoint were conducted using the same models described above, with additional terms of ESA administration and treatment by ESA interaction. The proportion of patients with RBC transfusions on/after week 5 was summarized by cycle for the first four cycles of treatment for each treatment group. To evaluate relationships among occurrence of grade 3/4 anemia, RBC transfusions, and ESA administration within a treatment group, Cohen's unweighted kappa was calculated to test concordance between two variables, and a chi‐square test performed to assess the association between them.

## 3 RESULTS

### 3.1 Patients

Overall, 242 patients were randomized (trilaciclib, *n* = 123; placebo, *n* = 119; ITT analysis set). As described previously, patient demographics and baseline disease characteristics were generally comparable but with slightly higher proportions of male patients and current smokers in the trilaciclib group than in the placebo group.^19^

### 3.2 Supportive care interventions for chemotherapy‐induced neutropenia

DSN in cycle 1 was significantly shorter in the trilaciclib group than in the placebo group (mean [SD], 0 [1.8] days vs. 4 [5.1] days; *p* < 0.0001). Throughout the treatment period, 14 patients (11.4%) in the trilaciclib group and 63 patients (52.9%) in the placebo group had SN (aRR [95% CI], 0.206 [0.120–0.351]; *p* < 0.0001).^19^

G‐CSF was administered to 28.5% of patients receiving trilaciclib, compared with 56.3% of patients receiving placebo (aRR [95% CI], 0.509 [0.371–0.700]; *p* < 0.0001).^19^ After accounting for G‐CSF administration, the effect of trilaciclib on the reduction of DSN in cycle 1 and occurrence of SN in cycle 1 remained statistically significant (both *p* < 0.0001). Across cycles 1 to 4, trilaciclib reduced mean DSN and occurrence of SN irrespective of G‐CSF administration (Table 2).

**TABLE 2:** Occurrence of SN and mean DSN in cycles 1 to 4, with or without G‐CSF administration

	G‐CSF administration			
	Yes		No	
	Trilaciclib	Placebo	Trilaciclib	Placebo
Occurrence of SN, *n*/*N* (%)				
Cycle 1^a^	2/12 (16.7)	20/25 (80.0)	6/108 (5.6)	38/92 (41.3)
*p* value^b^	0.0168		<0.0001	
Cycle 2^c^	2/17 (11.8)	10/39 (25.6)	2/92 (2.2)	11/68 (16.2)
*p* value^b^	0.1958		0.0022	
Cycle 3	1/17 (5.9)	7/44 (15.9)	2/79 (2.5)	8/54 (14.8)
Cycle 4	0/18 (0.0)	3/40 (7.5)	0/68 (0.0)	4/51 (7.8)
Mean (SD) DSN, days				
Cycle 1	0 (1.0)	7 (5.5)	0 (1.9)	4 (4.8)
*p* value^b^	0.0183		<0.0001	
Cycle 2	1 (1.6)	2 (5.2)	0 (0.7)	1 (3.0)
Cycle 3	0 (1.9)	1 (2.3)	0 (0.4)	2 (5.8)
Cycle 4	0 (0.0)	1 (3.4)	0 (0.0)	1 (1.9)

Abbreviations: DSN, duration of severe neutropenia; G‐CSF, granulocyte colony‐stimulating factor; SD, standard deviation; SN, severe neutropenia.

^a^ *p*_interaction_ = 0.7120.

^b^ Treatment group comparison for the patient subgroups.

^c^ *p_interaction_* = 0.2148.

Treatment effects for occurrence of SN in cycles 1 and 2 were statistically consistent between patients with or without G‐CSF administration (*p*_interaction_ = 0.7120 for cycle 1 and.2148 for cycle 2), and statistically significant reductions in DSN in cycle 1 and occurrence of SN were observed for patients with and without G‐CSF use (Table 2).

Although prophylactic G‐CSF was not permitted in cycle 1, it was used more often in the placebo group than in the trilaciclib group across cycles 2 to 4 (Table 3). The proportion of patients receiving therapeutic G‐CSFs was also higher in the placebo group in all cycles; few patients (<2%) in the trilaciclib group received therapeutic G‐CSFs in any cycle. Most G‐CSF use in the trilaciclib group was for “other” reasons (i.e., in the absence of a triggering neutropenia event).

**TABLE 3:** Reasons for G‐CSF administration in cycles 1 to 4 by treatment group (ITT analysis set)

Cycle Category, no. (%)	Trilaciclib (*n* = 123)	Placebo (*n* = 119)
Cycle 1^a^	120	117
Prophylactic	0 (0.0)	0 (0.0)
Therapeutic	2 (1.7)	19 (16.2)
Other	10 (8.3)	6 (5.1)
None	108 (90.0)	92 (78.6)
Cycle 2^a^	109	107
Prophylactic	2 (1.8)	21 (19.6)
Therapeutic	2 (1.8)	5 (4.7)
Other	12 (11.0)	13 (12.1)
None	93 (85.3)	68 (63.6)
Cycle 3^a^	96	98
Prophylactic	2 (2.1)	19 (19.4)
Therapeutic	1 (1.0)	4 (4.1)
Other	13 (13.5)	19 (19.4)
None	80 (83.3)	56 (57.1)
Cycle 4^a^	86	91
Prophylactic	2 (2.3)	20 (22.0)
Therapeutic	0 (0.0)	1 (1.1)
Other	15 (17.4)	17 (18.7)
None	69 (80.2)	53 (58.2)

Abbreviations: G‐CSF, granulocyte colony‐stimulating factor; ITT, intention‐to‐treat.

^a^ The number of patients who started the cycle is the denominator of percentages for each cycle.

### 3.3 Supportive care interventions for chemotherapy‐induced anemia

The proportion of patients with grade 3/4 anemia was significantly lower in the trilaciclib group than in the placebo group (20.3% vs. 31.9%; aRR [95% CI], 0.620 [0.405–0.949]; *p* = 0.0279). For patients receiving trilaciclib versus placebo, use of ESAs or RBC transfusions on/after week 5 was 3.3% versus 11.8% (*p* = 0.0254) and 14.6% versus 26.1% (*p* = 0.0252), respectively.^19^

**FIGURE 1:**
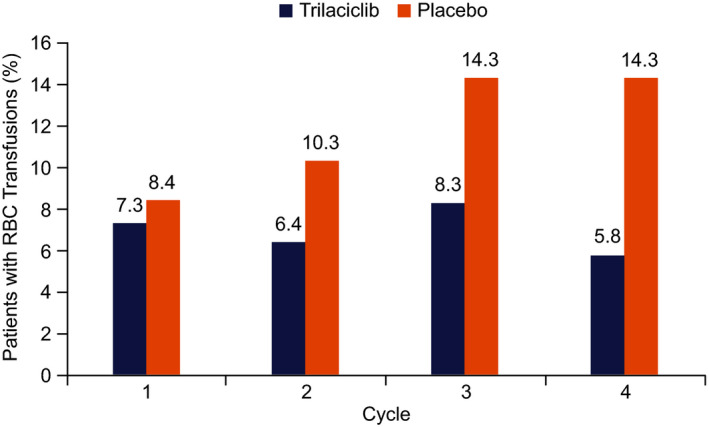
Occurrence of RBC transfusions in cycles 1 to 4 by treatment group. RBC indicates red blood cell

The proportion of patients with RBC transfusions was consistently lower in the trilaciclib group than in the placebo group at each cycle (Figure 1). RBC transfusions in the placebo group almost doubled over time (from 8.4% at cycle 1 to 14.3% at cycle 4), whereas the proportion of patients with RBC transfusions in the trilaciclib group remained relatively stable (range 5.8%–8.3%).

Among patients who did not receive ESAs, fewer patients in the trilaciclib group had grade 3/4 anemia or RBC transfusions on/after week 5 than in the placebo group (Table 4). Risk ratios could not be calculated because the number of patients who received ESAs was too low for statistical models to converge.

**TABLE 4:** Occurrence of grade 3/4 Anemia, and occurrence and event rate for RBC transfusions on/after week 5, with or without ESA administration

	ESA administration			
	Yes		No	
	Trilaciclib (*n* = 4)	Placebo (*n* = 14)	Trilaciclib (*n* = 119)	Placebo (*n* = 105)
Patients with grade 3/4 anemia, no. (%)	3 (75.0)	12 (85.7)	22 (18.5)	26 (24.8)
aRR (95% CI)	NE		0.693 (0.427–1.123)	
Patients with RBC transfusions on/after week 5, no. (%)	3 (75.0)	9 (64.3)	15 (12.6)	22 (21.0)
aRR (95% CI)	NE		0.577 (0.329–1.014)	
RBC transfusions on/after week 5, event rate per 100 weeks	10.9	7.8	1.2	2.6
aRR (95% CI)	NE		0.348 (0.174–0.696)	

Abbreviations: aRR, adjusted relative risk; CI, confidence interval; ESA, erythropoiesis‐stimulating agent; NE, not estimable; RBC, red blood cell.

Overall, there was high concordance between grade 3/4 anemia and RBC transfusions on/after week 5, regardless of treatment group. The chi‐square *p*‐value was significant (*p* < 0.0001 for both treatments), rejecting the hypothesis of no association. Concordance between grade 3/4 anemia and ESA administration was lower; however, the chi‐square *p*‐value was significant (trilaciclib, *p* = 0.0057; placebo, *p* < 0.0001). Among patients with grade 3/4 anemia, concordance between ESA administrations and RBC transfusions on/after week 5 was low (trilaciclib, *p* = 0.1661; placebo, *p* = 0.8969; Tables S2 and S3).

Overall, 49 patients (20.2%) had a total of 87 RBC transfusions on/after week 5. Of these, 43 patients (87.8%) had at least 1 RBC transfusion that was classified as clinically appropriate, and 11 patients (22.4%) had at least 1 transfusion event classified as inappropriate.

## 4 DISCUSSION

Neutropenia and anemia are common side effects of myelosuppressive chemotherapy that add to the total burden borne by patients with cancer and their families.^6^ In particular, for patients with severe or prolonged neutropenia, the likelihood of infection and serious consequences often necessitates hospitalization, as well as dose delays, dose reductions, and/or chemotherapy discontinuations that interfere with optimal treatment delivery.^20^

In this retrospective pooled analysis of data from three global, independent, randomized, double‐blind, placebo‐controlled, multicenter phase 2 trials in patients with ES‐SCLC, administering trilaciclib prior to chemotherapy significantly reduced DSN in cycle 1 and occurrence of SN across cycles 1 to 4 regardless of G‐CSF administration. Administering trilaciclib prior to chemotherapy significantly reduced the use of G‐CSF to approximately half that observed in patients receiving placebo. The reduction was apparent for both therapeutic and prophylactic administration. “Other” G‐CSF use, which was generally the largest category in the trilaciclib group, was more balanced between groups, suggesting that physicians frequently base G‐CSF use on subjective clinical judgment rather than objective data such as ANC counts and fever, with such cases occurring relatively evenly in both treatment groups.

Historically, consensus guidelines have recommended primary prophylactic use of G‐CSFs when the risk of FN is high (>20%) on the basis of chemotherapy and patient risk factors.^21^, ^22^ However, during the COVID‐19 pandemic, prophylactic G‐CSF use is also recommended where there is an intermediate (10%–20%) risk of FN.^23^ In the United States, most G‐CSF doses are administered during separate clinic visits, at least 24 h after administration of myelosuppressive chemotherapy, meaning that patients usually need to return to the clinic, often accompanied by a caregiver.^24^ Although self‐administration at home is an option, it can be undesirable due to high pharmacy co‐pays, patient discomfort with self‐treatment, and physician concerns.^24^ Therefore, overall, the reduced use of G‐CSFs with trilaciclib, together with the fact that trilaciclib is administered on the same day, prior to chemotherapy, has the potential to reduce the burden on health care systems, patients, and caregivers.

Despite the negative impact of chemotherapy‐induced anemia, its occurrence is frequently underestimated and its management is often delayed.^11^ Administering trilaciclib prior to chemotherapy significantly reduced both the occurrence and incidence of grade 3/4 anemia compared with placebo. There was also a significant reduction in the occurrence of ESA administration and in the use of RBC transfusions on/after week 5 in the trilaciclib group. The proportion of patients in each cycle who required RBC transfusions remained stable through successive cycles, likely reflecting protection of HSPCs continually exposed to cytotoxic chemotherapy. Conversely, RBC transfusions in the placebo group increased from cycle 1 to 4, suggesting that, in the absence of myeloprotection, the need for RBC transfusions increases with each cycle owing to cumulative HSPC damage.

Analyses of the occurrence of grade 3/4 anemia or the use of RBC transfusions by ESA administration were restricted by the limited number of patients who received ESAs. Among patients who did not receive ESAs, both the occurrence of grade 3/4 anemia and the use of RBC transfusions were significantly reduced with trilaciclib compared with placebo. For RBC transfusions, the interaction *p*‐value was below the statistical threshold for evidence of a differential treatment effect. However, the observed difference in RBC transfusions between patients who did or did not receive ESA administration should be regarded with caution given the very low patient numbers and number of ESA administrations. For grade 3/4 anemia, there was no differential treatment effect between patients who did or did not receive ESA administration.

There was a strong concordant relationship between RBC transfusions on/after week 5 and grade 3/4 anemia, indicating that most patients who received a transfusion had severe anemia and vice versa. Although grade 3/4 anemia did not frequently result in ESA administration, when ESAs were given, they were usually administered to patients with grade 3/4 anemia.

Although initial approval of ESAs in the United States reduced the need for RBC transfusions, subsequent studies suggested that ESA use was associated with decreased survival and/or tumor progression or recurrence, leading to changes in US prescribing practices.^25^ Furthermore, even in the responding patients, responses are often delayed.^26^ In parallel with the decreased use of ESAs, the requirement for RBC transfusions has increased; however, there has been a recent shift toward more restrictive use of RBC transfusions, with decreases in the threshold to 7.5 g/dl or even 7 g/dl.^25^ Considering the need to conserve limited blood supplies, many centers have adopted this lower threshold in the context of COVID‐19.^23^ By reducing the need for RBC transfusions and ESAs, trilaciclib may help to reduce the burden of chemotherapy‐induced anemia and potentially help to conserve limited blood supplies.

In addition to placing a considerable humanistic burden on patients and their caregivers,^6^ CIM and its management are associated with substantial economic costs.^27^, ^28^, ^29^ In a recent analysis of almost 350 patients with SCLC, 49% of patients received prophylactic or therapeutic G‐CSFs, 43% received RBC transfusions, and 4% received ESAs. Compared with patients without grade 3/4 hematologic events, incremental annual costs per patient for those with grade 3/4 hematologic adverse events were $63,245 for neutropenia and $28,152 for anemia.^28^ Similarly, an analysis in patients with metastatic breast cancer found that hematologic adverse events (particularly neutropenia and anemia) were the costliest chemotherapy‐related adverse events, with episodes involving hospitalization incurring the greatest financial costs.^29^ In a separate analysis of data pooled from the three trials of trilaciclib in patients with ES‐SCLC, 4.1% of patients receiving trilaciclib prior to chemotherapy were hospitalized due to CIM or sepsis, compared with 13.6% of patients receiving placebo (*p* = 0.0088).^19^ Together with the results from the current analysis, these data suggest that administering trilaciclib prior to chemotherapy has the potential to reduce the burden of CIM on health care systems by reducing the use of supportive care measures and CIM‐related hospital admissions.

## 5 CONCLUSIONS

The results from this pooled analysis show clear benefits associated with the administration of trilaciclib prior to chemotherapy in patients with ES‐SCLC. Compared with placebo, trilaciclib consistently reduced the duration and occurrence of SN, regardless of G‐CSF administration. This translated into a reduction in G‐CSF administration compared with placebo. Trilaciclib also consistently reduced the occurrence of chemotherapy‐induced anemia, which was reflected in the reduction of RBC transfusions on/after week 5 and ESA use. By improving key myelosuppressive endpoints and reducing the need for associated supportive care, trilaciclib has the potential to reduce both the societal and economic burden of CIM.

## CONFLICT OF INTEREST

Outside of the submitted work, Renata Ferrarotto has received personal fees from Ayala Pharma, Carevive, Cellestia Biotech, Klus Pharma, Medscape, Prelude, and Regeneron‐Sanofi, and has received grants from AstraZeneca, Genentech, Inc., Merck, Oropharynx Program Stiefel clinical trials, Pfizer, the ASCO Career Development Award, and the MD Anderson Khalifa Award; William Edenfield serves as a consultant for Chimerix Corp, and Jennifer M. Johnson has received research funding from AstraZeneca, Bristol Myers Squibb, and Merck, and has served as a consultant for Rakuten Medical and Foundation Medicine. Yili Pritchett and Rajesh K. Malik are employees and shareowners of G1 Therapeutics, Inc. Shannon R. Morris was an employee of G1 Therapeutics, Inc., at the time of study. Ian Anderson, Balazs Medgyasszay, Maria Rosario García‐Campelo, Trevor M. Feinstein, Sujith Kalmadi, Philip Lammers, A. Sanchez‐Hernandez, and Tibor Csőszi have no conflicts of interest to declare.

## AUTHOR CONTRIBUTIONS

Renata Ferrarotto: Investigation, writing—review and editing; Ian Anderson: Investigation, writing—review and editing; Balazs Medgyasszay: Investigation, writing—review and editing; Maria Rosario García‐Campelo: Investigation, writing—review and editing; William Edenfield: Investigation, writing—review and editing; Trevor M. Feinstein: Investigation, writing—review and editing; Jennifer M. Johnson: Investigation, writing—review and editing; Sujith Kalmadi: Investigation, writing—review and editing; Philip Lammers: Investigation, writing—review and editing; A. Sanchez‐Hernandez: Investigation, writing—review and editing; Yili Pritchett: Conceptualization, methodology, formal analysis, writing—review and editing; Shannon R. Morris: Conceptualization, methodology, formal analysis, writing—review and editing; Rajesh K. Malik: Conceptualization, methodology, formal analysis, writing—review and editing; Tibor Csőszi: Investigation, writing—review and editing.

## ETHICAL APPROVAL

All trials were designed and conducted in accordance with the Declaration of Helsinki and International Council for Harmonisation Good Clinical Practice guidelines. The protocols and study‐related materials were approved by the institutional review board or independent ethics committee of each participating site. All patients provided written informed consent.

## CONFLICT OF INTEREST

The authors declare that they have no competing interests.

## Supporting information

Table S1‐S3

## Data Availability

The data that support the findings of this study are available from the corresponding author upon reasonable request.
