# Supplementary material for: Trilaciclib prior to chemotherapy reduces the usage of supportive care interventions for chemotherapy‐induced myelosuppression in patients with small cell lung cancer: Pooled analysis of three randomized phase 2 trials
Source: Cancer Med. 2021 Aug 18;10(17):5748–56. doi: 10.1002/cam4.4089 (PMC8419768; doi:10.1002/cam4.4089)
Supplement: Supplementary file 1 — Table S1‐S3 [file CAM4-10-5748-s001.docx]

**Supporting Table 1.** Trial Populations and Treatments Administered in the Three Trials Included in the Pooled Analysis

| Trial ID | Population | Treatment Schedule (Randomized 1:1) |
| --- | --- | --- |
| G1T28-05 (NCT03041311) | First-line  ES-SCLC | Trilaciclib 240 mg/m^2^ IV QD or placebo prior to E/P/A^a^ on days 1-3 of each 21-day cycle for up to 4 cycles, followed by atezolizumab monotherapy (without trilaciclib/placebo) every 21 days |
| G1T28-02 (NCT02499770) | First-line ES-SCLC | Trilaciclib 240 mg/m^2^ IV QD or placebo prior to E/P^b^ on days 1-3 of each 21-day cycle |
| G1T28-03 (NCT02514447) | Second-/third-line ES-SCLC | Trilaciclib 240 mg/m^2^ IV QD or placebo prior to topotecan 1.5 mg/m^2^ IV QD on days 1-5 of each 21‑day cycle |

Abbreviations: AUC, area under the plasma concentration-time curve; E/P, etoposide/carboplatin; E/P/A, etoposide/carboplatin/atezolizumab; ES-SCLC, extensive-stage small cell lung cancer; ID, identification; IV, intravenous; QD, once daily.

In G1T28-02 and G1T28-03, trilaciclib or placebo prior to chemotherapy was administered until disease progression, unacceptable toxicity, withdrawal of consent, or discontinuation by the investigator. In G1T28-05, patients received up to 4 induction cycles with trilaciclib or placebo prior to E/P/A, followed by maintenance therapy with atezolizumab monotherapy. ^a^Standard-of-care IV etoposide (100 mg/m^2^) on days 1, 2, and 3, and carboplatin AUC 5 on day 1, with the addition of IV atezolizumab (1200 mg) on day 1 of each 21-day chemotherapy cycle.

^b^Standard-of-care IV etoposide (100 mg/m^2^) on days 1-3 and carboplatin AUC 5 on day 1, in 21-day cycles.

**Supporting Table 2.** Relationships Between Grade 3/4 Anemia, RBC Transfusions On/After Week 5, and ESA Administration (ITT Analysis Set)

|  | Trilaciclib (n = 123) | | Placebo (n = 119) | |
| --- | --- | --- | --- | --- |
|  | Patients With  Grade 3/4 Anemia  (n = 25) | Patients Without  Grade 3/4 Anemia  (n = 98) | Patients With  Grade 3/4 Anemia  (n = 38) | Patients Without  Grade 3/4 Anemia  (n = 81) |
| Patients with RBC transfusion on/after week 5, no. (%) | 16 (13.0) | 2 (1.6) | 29 (24.4) | 2 (1.7) |
| Patients without RBC transfusion on/after week 5, no. (%) | 9 (7.3) | 96 (78.0) | 9 (7.6) | 79 (66.4) |
| Chi-square *P*-value^a^ | <.0001 | | <.0001 | |
| Cohen’s kappa^b^ | .6917 | | .7764 | |
| Patients with ESA  administration, no. (%) | 3 (2.4) | 1 (0.8) | 12 (10.1) | 2 (1.7) |
| Patients with no ESA administration, no. (%) | 22 (17.9) | 97 (78.9) | 26 (21.8) | 79 (66.4) |
| Chi-square *P*-value^a^ | .0057 | | <.0001 | |
| Cohen’s kappa^b^ | .1598 | | .3497 | |

Abbreviations: ESA, erythropoiesis-stimulating agent; ITT, intention-to-treat; RBC, red blood cell.

^a^Calculated to test the association between two variables; the smaller the P-value, the stronger the evidence of an existing association.

^b^Measures the level of agreement between the two categories; the higher the values, the higher the concordance.

**Supporting Table 3.** Relationship Between ESA Administration and RBC Transfusions On/After Week 5 Among Patients with Grade 3/4 Anemia in Pooled Analysis Set

|  | Trilaciclib (n = 25) | | Placebo (n = 38) | |
| --- | --- | --- | --- | --- |
|  | Patients  With ESA Administration  (n = 3) | Patients Without ESA Administration  (n = 22) | Patients  With ESA Administration  (n = 12) | Patients Without ESA Administration  (n = 26) |
| Patients with RBC transfusion on/after week 5, no. (%) | 3 (12.0) | 13 (52.0) | 9 (23.7) | 20 (52.6) |
| Patients without RBC transfusion on/after week 5, no. (%) | 0 (0.0) | 9 (36.0) | 3 (7.9) | 6 (15.8) |
| Chi-square *P*-value^a^ | .1661 | | .8969 | |
| Cohen’s kappa^b^ | .1425 | | –.0139 | |

Abbreviations: ESA, erythropoiesis-stimulating agent; RBC, red blood cell.

^a^Calculated to test the association between two variables; the smaller the P-value, the stronger the evidence of an existing association.

^b^Measures the level of agreement between the two categories; the higher the values, the higher the concordance.
